# The differences in brain stem transcriptional profiling in hypertensive ISIAH and normotensive WAG rats

**DOI:** 10.1186/s12864-019-5540-5

**Published:** 2019-05-08

**Authors:** Larisa A. Fedoseeva, Leonid O. Klimov, Nikita I. Ershov, Vadim M. Efimov, Arcady L. Markel, Yuriy L. Orlov, Olga E. Redina

**Affiliations:** 1 Institute of Cytology and Genetics, Siberian Branch of Russian Academy of Sciences, Lavrentyeva, 10, Novosibirsk, Russian Federation 630090; 2 Novosibirsk State University, Novosibirsk, Russian Federation

**Keywords:** Stress-sensitive hypertension, Brain stem, Transcriptional profiling, RNA-Seq, ISIAH rat strain

## Abstract

**Background:**

The development of essential hypertension is associated with a wide range of mechanisms. The brain stem neurons are essential for the homeostatic regulation of arterial pressure as they control baroreflex and sympathetic nerve activity. The ISIAH (Inherited Stress Induced Arterial Hypertension) rats reproduce the human stress-sensitive hypertensive disease with predominant activation of the neuroendocrine hypothalamic-pituitary-adrenal and sympathetic adrenal axes. RNA-Seq analysis of the brain stems from the hypertensive ISIAH and normotensive control WAG (Wistar Albino Glaxo) rats was performed to identify the differentially expressed genes (DEGs) and the main central mechanisms (biological processes and metabolic pathways) contributing to the hypertensive state in the ISIAH rats.

**Results:**

The study revealed 224 DEGs. Their annotation in databases showed that 22 of them were associated with hypertension and blood pressure (BP) regulation, and 61 DEGs were associated with central nervous system diseases. In accordance with the functional annotation of DEGs, the key role of hormonal metabolic processes and, in particular, the enhanced biosynthesis of aldosterone in the brain stem of ISIAH rats was proposed. Multiple DEGs associated with several Gene Ontology (GO) terms essentially related to modulation of BP were identified. Abundant groups of DEGs were related to GO terms associated with responses to different stimuli including response to organic (hormonal) substance, to external stimulus, and to stress. Several DEGs making the most contribution to the inter-strain differences were detected including the *Ephx2,* which was earlier defined as a major candidate gene in the studies of transcriptional profiles in different tissues/organs (hypothalamus, adrenal gland and kidney) of ISIAH rats.

**Conclusions:**

The results of the study showed that inter-strain differences in ISIAH and WAG brain stem functioning might be a result of the imbalance in processes leading to the pathology development and those, exerting the compensatory effects. The data obtained in this study are useful for a better understanding of the genetic mechanisms underlying the complexity of the brain stem processes in ISIAH rats, which are a model of stress-sensitive form of hypertension.

**Electronic supplementary material:**

The online version of this article (10.1186/s12864-019-5540-5) contains supplementary material, which is available to authorized users.

## Introduction

Essential (primary) hypertension is a widely spread disease with underlying genetic predispositions, many of which still remain unknown. It was well documented that development of essential hypertension in humans is often associated with the elevated sympathetic nerve activity [1–3]. The increased sympathetic activity can contribute to sustained hypertension through its hemodynamic effects such as increased cardiac output and vascular resistance, as well as by altering renal sodium and water homeostasis [4]. The brain stem neurons control the sympathetic nerve activity [5] and play important role in regulation of arterial pressure [6–9].

For better understanding of the molecular basis underlying the brain mechanisms initiating essential hypertension, a number of animal models are widely used [10, 11]. These studies underscore the complexity of genetic mechanisms involved in the blood pressure (BP) regulation and point out that phenotypic appearance may depend on the differences in genetic background and/or physiological conditions.

The ISIAH (Inherited Stress Induced Arterial Hypertension) rat strain is a model of hypertension with the genetically determined enhanced responsiveness to stressful stimulation [12]. The ISIAH rat strain was selected from Wistar rats for enhanced response of the systolic arterial BP to a mild emotional stress caused by 30 min restraint in a cylindrical wire-mesh cage [13, 14]. Adult ISIAH rats are characterized by elevated of both the basal and the stress-induced arterial BP. Using the fingerprinting method, it was shown that ISIAH rats are an inbred strain, which makes them an adequate model for studying the molecular genetic basis of the stress-sensitive form of hypertension [15].

One of the important ways to ascertain the genetic determination of hypertension development is to study the transcriptional activity of individual genes and the genome as a whole [16, 17]. The next-generation sequencing technologies are currently widely used to produce an analysis of the entire transcriptomes (RNA-Seq approach) and to identify the molecular mechanisms underlying the complex diseases development [18].

The aim of the current work was to perform the comparative analysis of brain stem transcriptomes in hypertensive ISIAH and normotensive WAG (Wistar Albino Glaxo) rats and to reveal the differentially expressed genes (DEGs), which may play the key role in brain stem functioning, and metabolic pathways contributing to the stress-sensitive hypertension.

## Results

### The inter-strain gene expression differences

Altogether, 13,546 genes were defined as transcribed in brain stems of ISIAH and WAG male rats and were used in further analysis, which revealed 224 DEGs. These genes are listed in the Additional file 1: Table S1. Their hierarchical clustering based on Euclidean distance is presented in Additional file 2: Figure S2. The majority of DEGs (57.1%) were downregulated in ISIAH rats.

Transcription of 9 genes was found in the brain stem of one rat strain and not detected in the brain stem of another rat strain (Additional file 3: Table S3). Most of these genes are poorly studied and none of them is currently associated with hypertension development.

### Functional annotation of DEGs

**Fig. 1:**
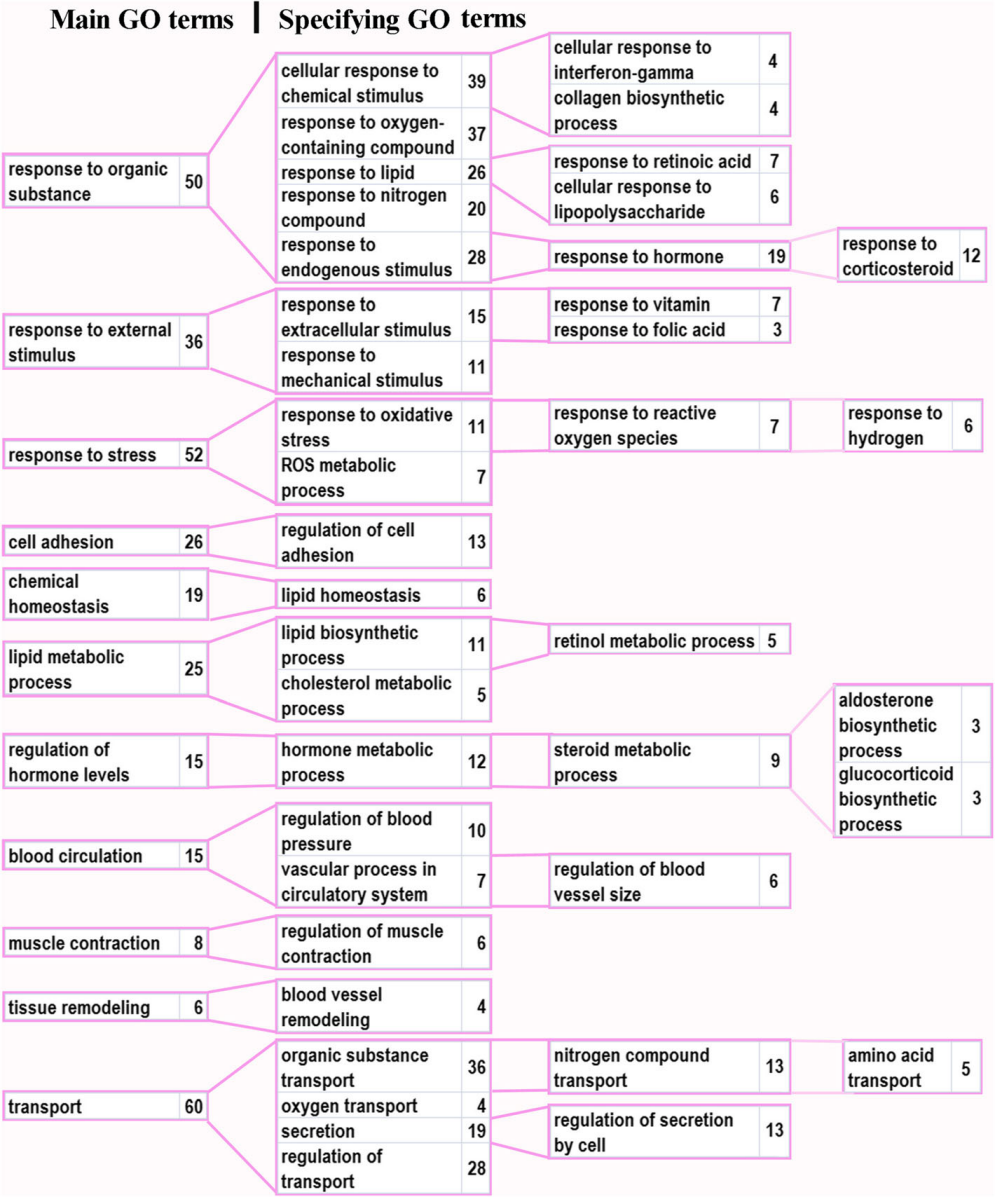
Gene Ontology (GO) terms specifying the main GO terms. Numerals represent the number of genes in the group

The significantly (*p* < 0.05) enriched GO terms for biological processes, which may have relation to hypertensive state of ISIAH rats are shown in Additional file 4: Figure S4. The genes associated with those GO terms are listed in Additional file 5: Table S5. According to the sets of genes associated with the biological processes taken into consideration, the main GO terms and subgroups describing the specificity of the main processes were detected (Fig. 1).

**Fig. 2:**
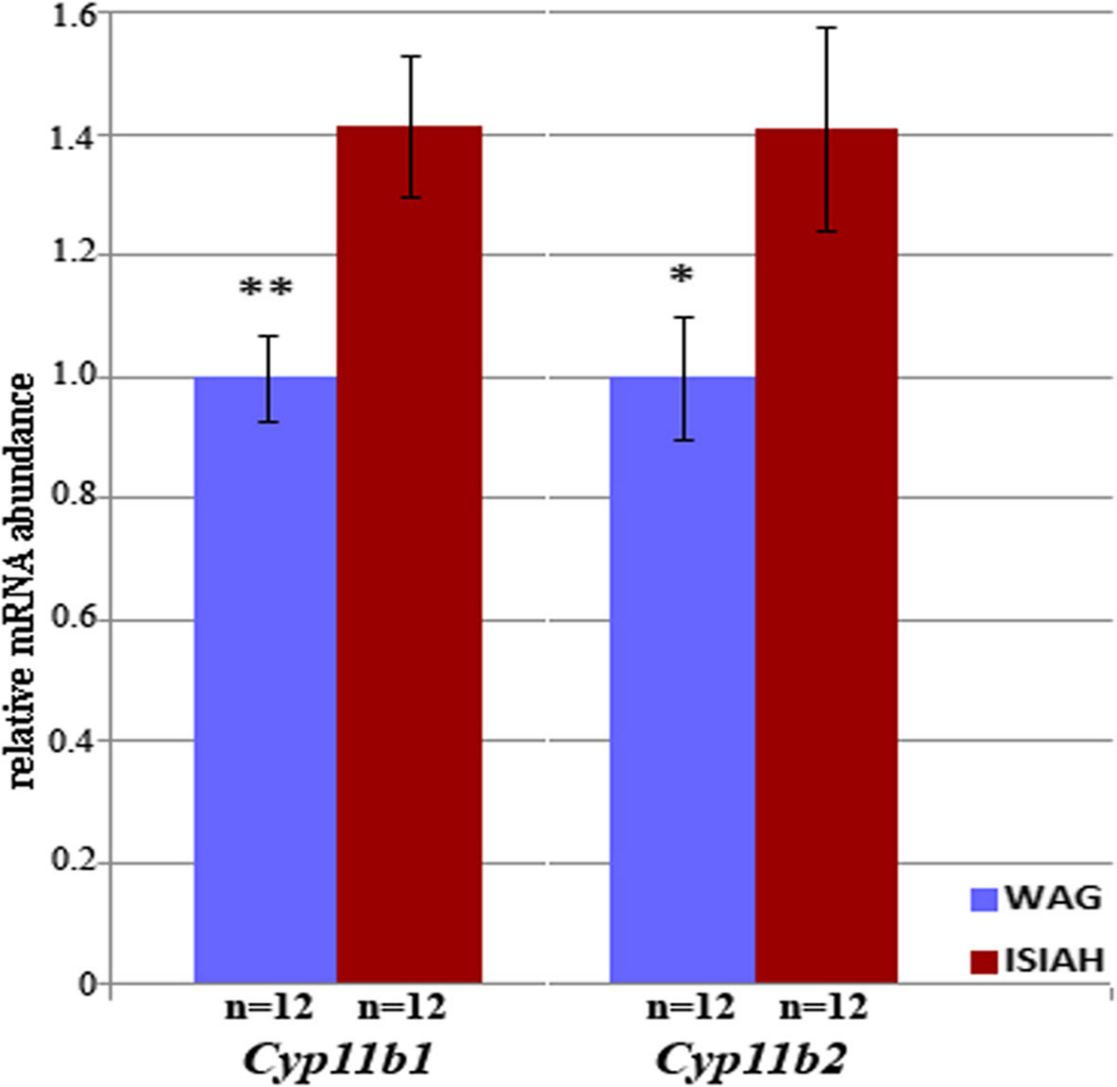
qPCR measurement of mRNA for the *Cyp11b1* and *Cyp11b2* genes encoding for the 11β-hydroxylase and aldosterone synthase enzymes, respectively, in brain stem of ISIAH and WAG rats. The significance of inter-strain difference is indicated by * *p* < 0.05; ** *p* < 0.01

The most significant enrichment of GO term ‘hormone metabolic process’ (Additional file 4: Figure S4) underlined the importance of the hormone levels regulation in the stress-sensitive hypertension development. The GO term ‘hormone metabolic process’ included the genes related to the steroid metabolic process, particularly to aldosterone and glucocorticoid biosynthetic processes. Key DEGs involved in these biosynthetic processes (*Cyp11a1, Cyp11b1, Cyp11b2*) were upregulated in brain stem of hypertensive ISIAH rats. However, the transcription levels of *Cyp11b1* and *Cyp11b2* were tested together by Cufflinks/Cuffdiff programs, possibly because of high similarity in their mRNA sequences. The quantitative real time PCR (qPCR) analysis performed to distinguish between the levels of their expression confirmed the elevated transcription of both genes in the brain stem of ISIAH rats (Fig. 2). The enzymes encoded by *Cyp11b1* and *Cyp11b2* are key players in many biological processes including glucocorticoid (corticosterone) and aldosterone biosynthetic processes. Besides, GO enrichment analysis showed that BP in ISIAH rats may be modulated by multiple DEGs associated with several other GO terms essentially related to BP regulation. These are ‘blood circulation’, ‘regulation of BP’, ‘regulation of blood vessel size’, ‘regulation of muscle contraction’ and ‘blood vessel remodeling’ (Additional file 5: Table S5).

Many GO terms were associated with responses to different stimuli. Among these, the most abundant groups of DEGs were related to ‘response to organic substance’, ‘response to external stimulus’, and ‘response to stress’. The specifying GO terms underlined the importance of such processes as responses to interferon-gamma, to retinoic acid, to lipopolysacchride, to hormone (corticosteroid), to vitamin and folic acid, and to reactive oxygen species (ROS) (Fig. 1). Multiple DEGs were also associated with transport/secretion and regulation of transport. These and some other groups of DEGs associated with ‘cell adhesion’, ‘chemical homeostasis’, ‘lipid metabolic process’ pointed out the processes contributing to multiple impairments in functioning of brain stem in hypertensive ISIAH rats.

**Fig. 3:**
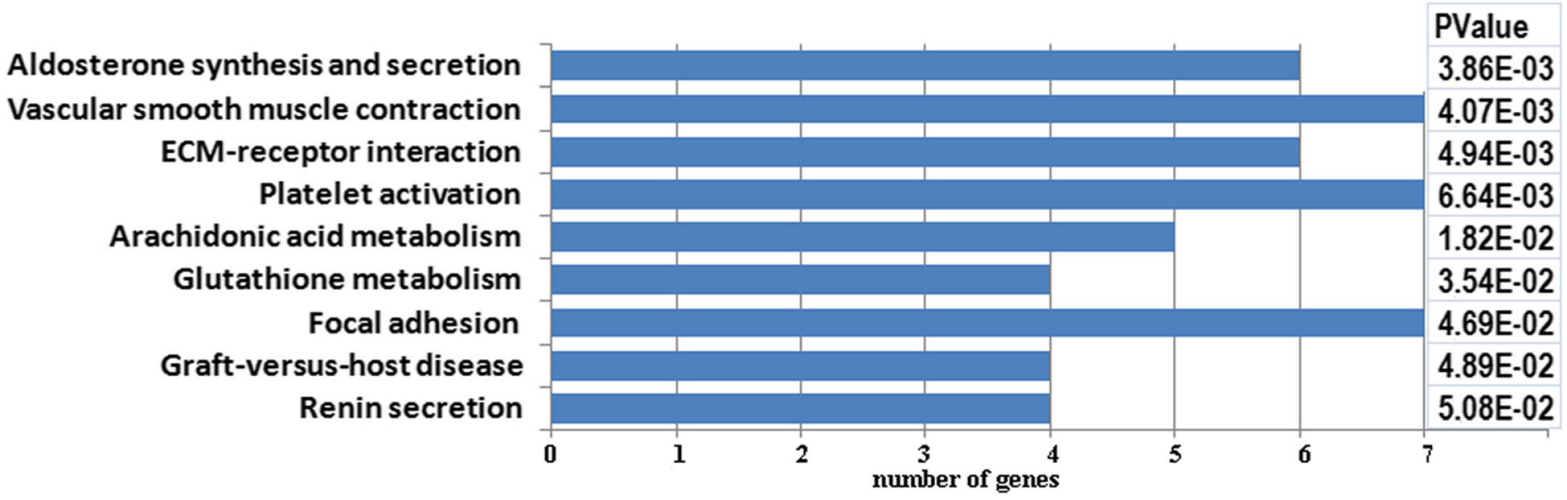
The significantly enriched (*p* < 0.05) KEGG (Kyoto Encyclopedia of Genes and Genomes) pathways

Eight significantly enriched (*p* < 0.05) KEGG (Kyoto Encyclopedia of Genes and Genomes) pathways were identified in the current study (Fig. 3). The genes associated with these KEGG terms are listed in Additional file 6: Table S6. Similarly to the results of the functional annotation in Gene Ontology Database, the most significantly enriched pathways were associated with ‘aldosterone synthesis and secretion’ and ‘vascular smooth muscle contraction’.

### Genes associated with hypertension and BP regulation

The annotation of DEGs in RGD (Rat Genome Database) revealed 18 genes associated with hypertension, and according to DAVID, four additional DEGs (*Acta2, Hba2, P2rx4*, and *Sult1a1*) were defined as related to regulation of BP. These 22 DEGs may be considered as candidates contributing to hypertension development in ISIAH rats (Table 1). Almost all of these genes (19 of them) are associated with CNS (central nervous system) diseases. Three of them (*Ephx2, F2r* and *Il18*) are known as contributing to brain ischemia. All KEGG metabolic pathways reported in Additional file 6, Table S6 contain genes associated with hypertension.

**Table 1:** Genes differentially expressed in ISIAH and WAG brain stems and referred to in Databases as associated with blood pressure regulation and hypertension

Gene symbol	Gene_ID	log2 (fold_change) ISIAH/WAG	Gene definition
Rat Genome Database			
Aoc3*	29473	−1.40	amine oxidase, copper containing 3 (vascular adhesion protein 1)
Cbs*	24250	0.54	cystathionine beta synthase
Chi3l1*	89824	−1.36	chitinase 3-like 1 (cartilage glycoprotein-39)
Col1a1*	29393	−0.73	collagen, type I, alpha 1
Col3a1*	84032	−1.08	collagen, type III, alpha 1
Cyp11a1*	29680	2.55	cytochrome P450, family 11, subfamily a, polypeptide 1
*Cyp11b1*	500892	1.12	cytochrome P450, family 11, subfamily b, polypeptide 1
Cyp11b2*	24294	1.12	cytochrome P450, family 11, subfamily b, polypeptide 2
Ephx2* ^**#**^	65030	4.23	epoxide hydrolase 2, cytoplasmic
F2r* ^**#**^	25439	0.81	coagulation factor II (thrombin) receptor
Fn1*	25661	−1.30	fibronectin 1
Gstm2*	24424	−1.10	glutathione S-transferase mu 2
Gucy1a3*	497757	−0.69	guanylate cyclase 1, soluble, alpha 3
Igfbp2*	25662	−0.51	insulin-like growth factor binding protein 2
Il18* ^**#**^	29197	−1.29	interleukin 18
*Npr1*	24603	−0.61	natriuretic peptide receptor A/guanylate cyclase A (atrionatriuretic peptide receptor A)
Ptgds*	25526	−0.74	prostaglandin D2 synthase (brain)
Tlr3*	364594	0.96	toll-like receptor 3
DAVID			
Acta2*	81633	−0.74	smooth muscle alpha-actin
*Hba2*	360504	0.58	hemoglobin alpha 2 chain
P2rx4*	29659	−0.88	purinergic receptor P2X, ligand-gated ion channel 4
Sult1a1*	83783	−0.85	sulfotransferase family, cytosolic, 1A, phenol-preferring, member 1

Genes associated with *- central nervous system diseases; ^**#**^ - brain ischemia;

ISIAH and WAG – rat strains used in the study

### Genes associated with CNS diseases

Altogether, there were 61 DEGs referred to in RGD as associated with CNS diseases (Table 2). Eight of them may contribute to brain ischemia. The association of these DEGs with GO terms for biological processes is shown in Additional file 5: Table S5. The KEGG pathways-associated DEGs are shown in Additional file 6, Table S6.

**Table 2:** Genes differentially expressed in ISIAH and WAG brain stems and referred to in Rat Genome Database as associated with central nervous system diseases

Gene symbol	Gene_ID	log2 (fold_change) ISIAH/WAG	Gene definition
*Abcg2*	312382	1.84	ATP-binding cassette, subfamily G (WHITE), member 2
*Acadsb*	25618	−0.61	acyl-CoA dehydrogenase, short/branched chain
*Acta2*	81633	−0.74	smooth muscle alpha-actin
*Aldh1a1*	24188	0.51	aldehyde dehydrogenase 1 family, member A1
*Aldh1a2*	116676	−0.67	aldehyde dehydrogenase 1 family, member A2
*Aoc3*	29473	−1.40	amine oxidase, copper containing 3 (vascular adhesion protein 1)
*Atp10a*	365266	−0.75	ATPase, class V, type 10A
*Bmp6* ^**#**^	25644	−0.65	bone morphogenetic protein 6
*Cadps2*	312166	0.56	Ca++ − dependent secretion activator 2
*Car8*	297814	1.29	carbonic anhydrase 8
*Casp4*	114555	1.07	caspase 4, apoptosis-related cysteine peptidase
*Cbs*	24250	0.54	cystathionine beta synthase
*Cckbr*	25706	−1.25	cholecystokinin B receptor
*Chi3l1*	89824	−1.36	chitinase 3-like 1 (cartilage glycoprotein-39)
*Col1a1*	29393	−0.73	collagen, type I, alpha 1
*Col3a1*	84032	−1.08	collagen, type III, alpha 1
*Col6a3*	367313	−0.87	procollagen, type VI, alpha 3
*Cyp11a1*	29680	2.55	cytochrome P450, family 11, subfamily a, polypeptide 1
*Cyp11b2*	24294	1.12	cytochrome P450, family 11, subfamily b, polypeptide 2
*Des*	64362	−0.90	desmin
*Eif3c*	293484	−0.52	eukaryotic translation initiation factor 3, subunit C
*Ephx2* ^**#**^	65030	4.23	epoxide hydrolase 2, cytoplasmic
*Ercc2*	308415	−2.96	excision repair cross-complementing rodent repair deficiency, complementation group 2
*F2r* ^**#**^	25439	0.81	coagulation factor II (thrombin) receptor
*Fhit*	60398	2.12	fragile histidine triad gene
*Flna*	293860	−0.58	filamin A, alpha
*Fn1*	25661	−1.30	fibronectin 1
*Gabra6* ^**#**^	29708	3.65	gamma-aminobutyric acid (GABA) A receptor, alpha 6
*Gabrd*	29689	1.22	gamma-aminobutyric acid (GABA) A receptor, delta
*Grm2*	24415	−2.74	glutamate receptor, metabotropic 2
*Gstm2*	24424	−1.10	glutathione S-transferase mu 2
*Gucy1a3*	497757	−0.69	guanylate cyclase 1, soluble, alpha 3
*Hoxb8*	24457	1.04	homeo box B8
*Igf2* ^**#**^	24483	−0.93	insulin-like growth factor 2
*Igfbp2*	25662	−0.51	insulin-like growth factor binding protein 2
*Il16*	116996	1.22	interleukin 16
*Il18* ^**#**^	29197	−1.29	interleukin 18
*Itpr1*	25262	0.79	inositol 1,4,5-trisphosphate receptor, type 1
*Itpripl1*	499885	1.36	inositol 1,4,5-trisphosphate receptor interacting protein-like 1
*Ltbp1* ^**#**^	59107	−0.72	latent transforming growth factor beta binding protein 1
*Mal*	25263	−0.78	mal, T-cell differentiation protein
*Mapre2*	679221	−1.01	microtubule-associated protein RP/EB family member 2-like
*Mccc2*	361884	−0.60	methylcrotonoyl-CoA carboxylase 2 (beta)
*Mcm7*	288532	0.80	minichromosome maintenance complex component 7
*Mgmt*	25332	−0.96	O-6-methylguanine-DNA methyltransferase
*Myh11*	24582	−0.51	myosin, heavy chain 11, smooth muscle
*Ncaph*	680089	2.40	non-SMC condensin I complex, subunit H
*Nptx2*	288475	−1.35	neuronal pentraxin II
*P2rx4*	29659	−0.88	purinergic receptor P2X, ligand-gated ion channel 4
*P2ry12*	64803	0.66	purinergic receptor P2Y, G-protein coupled, 12
*Pld5*	289270	−0.99	phospholipase D family, member 5
*Ppm1k*	312381	−0.52	protein phosphatase, Mg2+/Mn2+ dependent, 1 K
*Ptgds*	25526	−0.74	prostaglandin D2 synthase (brain)
*Rbp4*	25703	−1.48	retinol binding protein 4, plasma
*Retsat*	246298	1.86	retinol saturase (all trans retinol 13,14 reductase)
*Snx14*	315871	−0.66	sorting nexin 14
*Sult1a1*	83783	−0.85	sulfotransferase family, cytosolic, 1A, phenol-preferring, member 1
*Tlr3*	364594	0.96	toll-like receptor 3
*Ttr* ^**#**^	24856	−2.80	transthyretin
*Vtn*	29169	−0.55	vitronectin
*Zbtb16*	353227	−0.84	zinc finger and BTB domain containing 16

^#^- genes associated with brain ischemia; ISIAH and WAG – rat strains used in the study

### Transcription factor genes

Thirteen transcription factor genes were differentially expressed in ISIAH and WAG brain stem structures (Table 3). Several of them are referred to in RGD as associated with CNS diseases. The transcription factor genes differentially expressed in ISIAH and WAG brain stems are associated with many GO terms for biological processes (Additional file 5: Table S5). One of them (*Star*) is associated with ‘aldosterone synthesis and secretion’ pathway.

**Table 3:** Transcription factor genes differentially expressed in ISIAH and WAG brain stems

Gene symbol	Gene_ID	log2 (fold_change) ISIAH/WAG	Gene definition
*Aebp1*	305494	−0.52	AE binding protein 1
Ercc2*	308415	−2.96	excision repair cross-complementing rodent repair deficiency, complementation group 2
*Grhl3*	298555	1.37	grainyhead-like 3 (Drosophila)
Hoxb8*	24457	1.04	homeo box B8
*Irf7*	293624	1.02	interferon regulatory factor 7
Mal*	25263	−0.78	mal, T-cell differentiation protein
Mcm7*	288532	0.80	minichromosome maintenance complex component 7
*Neurod1*	29458	2.10	neurogenic differentiation 1
*Nfkbil1*	361794	0.96	nuclear factor of kappa light polypeptide gene enhancer in B-cells inhibitor-like 1
*Pbx3*	311876	−1.43	pre-B-cell leukemia homeobox 3
*Star*	25557	2.49	steroidogenic acute regulatory protein
Zbtb16*	353227	−0.84	zinc finger and BTB domain containing 16
*Zfp488*	290571	−1.26	zinc finger protein 488

*- genes associated with central nervous system diseases (according to the RGD annotation);

ISIAH and WAG – rat strains used in the study

### Genes defining the most inter-strain differences

**Fig. 4:**
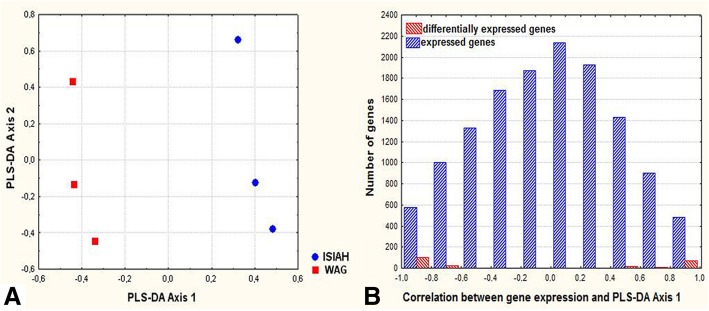
**a** Axes maximizing the distances between ISIAH and WAG rats; **b** The distribution of expressed genes along the axis representing the correlation between gene expression and PLS-DA Axis 1

The PLS-DA (partial-least squares discriminant analysis) was employed to reveal the genes with the most impact to inter-strain differences. The axes maximizing the distances between ISIAH and WAG rats were constructed (Fig. 4a) and the Pearson correlation between gene expression and PLS-DA Axis 1 was calculated. The distribution of the genes along the axis representing the correlation between gene expression and PLS-DA Axis 1 is shown in Fig. 4b. Genes settled to the polar position in the histogram may be considered as genes defining the most inter-strain differences. DEGs are given in red in Fig. 4b and their polar position along the axis underlines their high impact to the existing inter-strain variations.

**Fig. 5:**
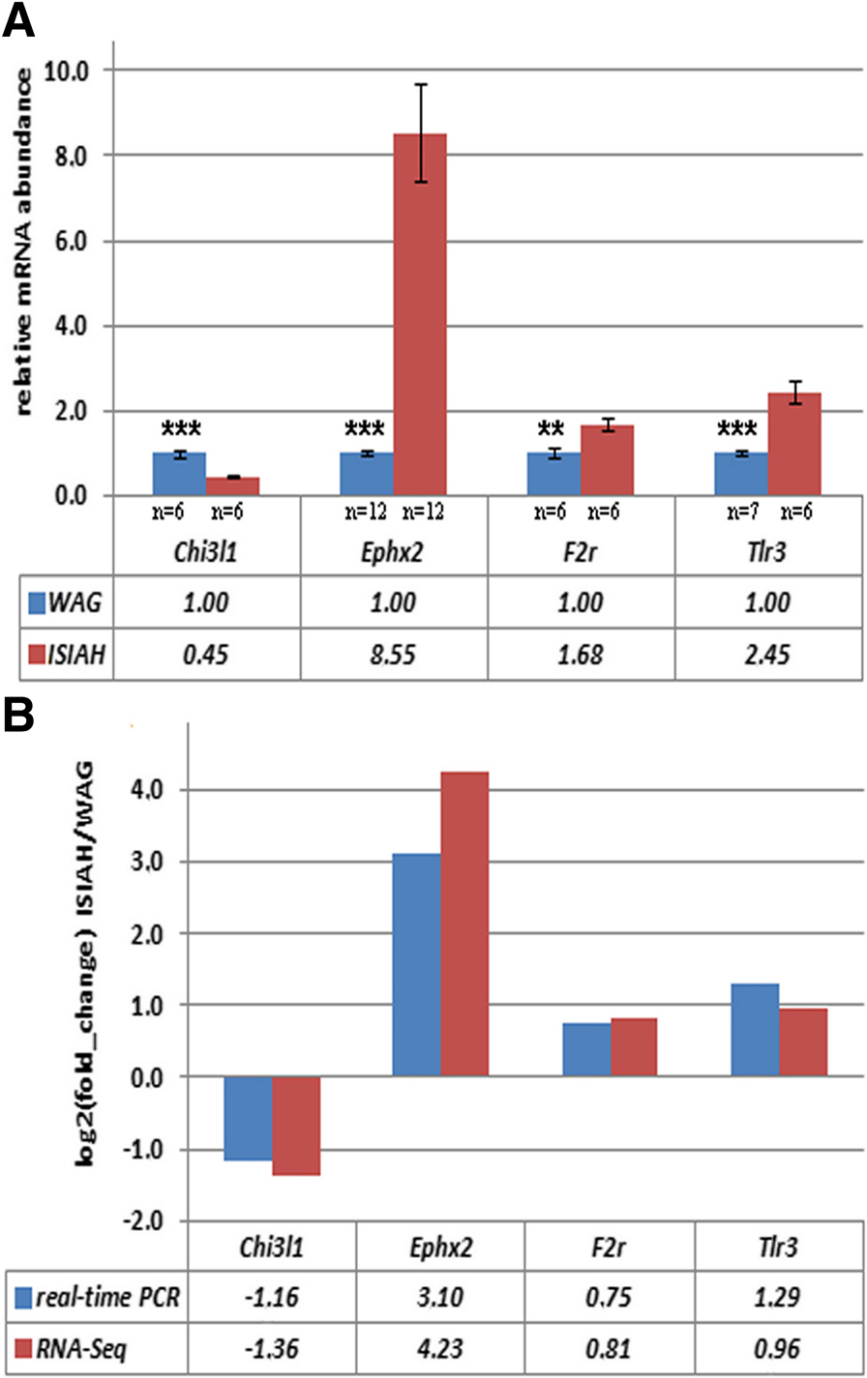
**a** The relative mRNA abundance measured by qPCR. **b** The comparison of the relative mRNA abundance between the RNA-Seq and qPCR measurements. The significance of inter-strain difference is indicated by **p < 0.01, ***p < 0.001

Top 50 DEGs characterized by the highest correlation coefficients between gene expression and PLS-DA Axis 1 are listed in Table 4. Differential transcription of four of these DEGs, which are known as associated with hypertension (*F2r, Chi3l1, Ephx2*, and *Tlr3*), was validated by qPCR (Fig. 5a). Estimation of the genes’ expression by RNA-Seq and qPCR showed highly similar results, with a calculated correlation coefficient of 0.98 between the two methods (Fig. 5b).

**Table 4:** The top 50 DEGs making the most significant contribution to the inter-strain differences

Gene symbol	Gene ID	r	log2 (fold_change) ISIAH/WAG	Gene definition
*Mpeg1*	64552	−0.998	−3.11	macrophage expressed 1
*RT1-A2*	24974	−0.995	−1.94	RT1 class Ia, locus A2
*Gstm4*	499689	−0.994	−1.95	glutathione S-transferase mu 4
*Mettl24*	499465	−0.993	expressed only in WAG rat strain	methyltransferase like 24
*Grm2*	24415	−0.993	−2.74	glutamate receptor, metabotropic 2
*Rufy1*	360521	−0.992	−0.62	RUN and FYVE domain containing 1
*LOC688584*	688584	−0.992	expressed only in WAG rat strain	similar to BCL2/adenovirus E1B 19 kDa-interacting protein 3-like
*RT1-S3*	294228	−0.992	−2.02	RT1 class Ib, locus S3
*Slc25a48*	361206	−0.991	−1.77	solute carrier family 25, member 48
*LOC100360731*	100360731	−0.991	expressed only in WAG rat strain	SNRPN upstream reading frame protein-like
*LOC678760*	678760	−0.989	expressed only in WAG rat strain	dipeptidyl peptidase 3-like
*Eif3c*	293484	−0.988	−0.52	eukaryotic translation initiation factor 3, subunit C
*Fn1*	25661	−0.986	−1.30	fibronectin 1
*Cryab*	25420	−0.986	−0.87	crystallin, alpha B
*Abca8a*	303638	−0.986	−0.85	ATP-binding cassette, subfamily A (ABC1), member 8a
*Nptx2*	288475	−0.984	−1.35	neuronal pentraxin II
Chi3l1*	89824	−0.983	−1.36	chitinase 3-like 1 (cartilage glycoprotein-39)
*Ly75*	499800	−0.983	−0.79	lymphocyte antigen 75
*Mx2*	286918	−0.981	−1.49	myxovirus (influenza virus) resistance 2
*Sdf2l1*	680945	−0.981	−0.96	stromal cell-derived factor 2-like 1
*Grifin*	117130	−0.980	−5.85	galectin-related inter-fiber protein
*Pld5*	289270	−0.978	−0.99	phospholipase D family, member 5
*Tmem119*	304581	−0.977	−1.43	transmembrane protein 119
*Lcat*	24530	−0.976	−1.20	lecithin cholesterol acyltransferase
*Pla2g3*	289733	−0.976	−0.84	phospholipase A2, group III
*Pnpla1*	361812	−0.976	−2.94	patatin-like phospholipase domain containing 1
*Flnc*	362332	−0.975	−0.64	filamin C, gamma
*Neu4*	316642	−0.973	−0.96	sialidase 4
F2r*	25439	0.973	0.81	coagulation factor II (thrombin) receptor
*Enpp6*	306460	0.973	1.53	ectonucleotide pyrophosphatase/phosphodiesterase 6
*LOC100134871*	100134871	0.977	3.65	beta globin minor gene
*LOC100362027*	100362027	0.979	expressed only in ISIAH rat strain	ribosomal protein L30-like
*Abhd1*	313917	0.980	1.97	abhydrolase domain containing 1
*RGD1564278*	312994	0.980	expressed only in ISIAH rat strain	RNA-binding protein with serine-rich domain 1-like
*Golm1*	680692	0.980	0.63	Golgi membrane protein 1-like
*Leprel4*	59101	0.981	1.00	leprecan-like 4
*RGD1565131*	498143	0.981	7.05	60S ribosomal protein L15-like
*LOC688504*	688504	0.981	expressed only in ISIAH rat strain	60S ribosomal protein L28 pseudogene
*Retsat*	246298	0.982	1.86	retinol saturase (all trans retinol 13,14 reductase)
Ephx2*	65030	0.982	4.23	epoxide hydrolase 2, cytoplasmic
*Ndufa10l1*	316632	0.983	1.63	NADH dehydrogenase (ubiquinone 1 alpha subcomplex 10-like 1
*Evc*	289712	0.986	1.03	Ellis van Creveld syndrome homolog (human)
*Sc5dl*	114100	0.987	1.24	sterol-C5-desaturase (ERG3 delta-5-desaturase homolog, S, cerevisiae)-like
*Sacm2l-ps3*	415066	0.988	expressed only in ISIAH rat strain	Sacm2l-ps3 pseudogene
Tlr3*	364594	0.989	0.96	toll-like receptor 3
*Fhit*	60398	0.991	2.12	fragile histidine triad gene
*Abcg2*	312382	0.991	1.84	ATP-binding cassette, subfamily G (WHITE), member 2
*LOC685744*	685744	0.993	expressed only in ISIAH rat strain	hypothetical protein LOC685744
*LOC360998*	360998	0.998	0.90	hypothetical LOC360998
*Fhad1*	500577	0.999	2.31	forkhead-associated (FHA) phosphopeptide binding domain 1

*- genes associated with hypertension; ISIAH and WAG – rat strains used in the study

r - correlation coefficient between gene expression and PLS-DA Axis 1

## Discussion and conclusions

The brain stem neurons are essential for the homeostatic regulation of arterial pressure as they control baroreflex and sympathetic nerve activity. The results of the current study helped to assess the transcriptional profiles of the brain stems in hypertensive ISIAH and normotensive WAG rats and to detect the DEGs. These data were subjected to functional analysis to identify the genes, which may contribute to the stress-sensitive hypertension.

The functional annotation of genes differentially transcribed in the brain stems of compared rat strains underlined the importance of the hormone (steroids) levels regulation including aldosterone and glucocorticoid biosynthetic processes. The DEGs involved in these steroid biosynthetic processes are known as key players in steroidogenic pathways [19]. Their upregulation in the brain stem of ISIAH rats suggests the increase in steroid hormones production, particularly aldosterone synthesis. According to the pathway analysis in the KEGG database, the ‘aldosterone synthesis and secretion’ pathway was also determined as the most significantly enriched.

The enhanced brain aldosterone may upregulate the sympathetic nervous activity [20]. It was demonstrated that the small amounts of aldosterone synthesized in the brain of Dahl (SS) rats could activate the mineralocorticoid receptors [21], and that process was important in the genesis of the salt-sensitive hypertension [22]. It was shown that aldosterone synthesis in the brain may contribute to salt-sensitive hypertension through the elevated salt appetite, increased sympathetic drive and vasopressin secretion [23].

Our results of qPCR obtained for *Cyp11b1* and *Cyp11b2* expression (Fig. 2) are similar to those described in the study of aldosterone synthesis in brain stems from SS rats with salt-sensitive hypertension [22]. The increased transcription of the *Cyp11b1* and *Cyp11b2* genes involved in the steroid biosynthesis in ISIAH brain stem suggests that elevated level of aldosterone synthesis may have an impact to the development of stress-sensitive hypertension, too.

It has been long known that the regulation of the vascular resistance in the brain stem plays an important role in the autoregulation of the brain blood flow [24]. In the current study, the ‘vascular smooth muscle contraction’ pathway was one of the most significantly enriched (Fig. 3). Altered expression of the DEGs associated with this metabolic pathway may exert different effects on vascular smooth muscle (VSM) contraction process. Thus, the transcription of *Itpr1* encoding inositol 1,4,5-trisphosphate receptor, type 1 (IP_3_R) was increased. IP_3_R up-regulation in VSM is associated with enhancement and sensitization of IP_3_-dependent Ca^2+^ release, resulting in increased VSM contraction in response to agonist stimulation [25]. The decreased level of *Gucy1a3* and *Npr1* transcription may also contribute to increased VSM contraction in the brain stem of ISIAH rats, as *Gucy1a3* encodes for soluble guanylate cyclase 1, alpha 3, which functions as an intracellular nitric oxide (NO) receptor and contributes to vascular smooth muscle relaxation [26] and *Npr1* encodes a receptor for atrial and brain natriuretic peptides [27] known as a potent aldosterone antagonists and vasodilators. Alternatively, the downregulation of *Acta2* encoding contractile protein [28] may be protective against excessive smooth muscle contraction in ISIAH rats.

The annotation of DEGs in Gene Ontology database also revealed several groups of genes essentially related to modulation of BP and hypertension development (‘blood circulation’, ‘regulation of BP’, ‘regulation of blood vessel size’, ‘regulation of muscle contraction’ and ‘blood vessel remodeling’). Many DEGs in these groups are referred to in Rat Genome Database as associated with hypertension. So, the genes in these groups may be considered as candidate genes contributing to the mechanisms of blood flow and BP regulation in the brain stem of ISIAH rats.

The ISIAH rats were previously characterized as a model of hypertension with the predominant involvement of the hypothalamic-pituitary-adrenal and sympathetic adrenal medullary systems [12]. The results of the current study suggest the existence of multiple impairments in brain stem functioning in hypertensive ISIAH rats. The functional annotation revealed several abundant groups of DEGs associated with responses to different stimuli: to organic substance, to external stimulus, and to stress. As can be seen from Fig. 1, the response to organic substance is associated with various stimuli, the most important of which is probably the endogenous (hormonal) stimulus. The central role of the enhanced steroid biosynthesis in ISIAH brain stem has already been discussed above, so it was very much expected that a set of genes associated with the response to corticosteroids was discovered.

Another abundant group of DEGs was associated with response to stress. Multiple DEGs in this group are known as related to response to oxidative stress and ROS metabolic processes. The oxidative stress in brain stem sites (nucleus tractus solitarii (NTS) and rostral ventrolateral medulla (RVLM)) engaged in the regulation of sympathetic vasomotor tone is known as one of the underlying mechanisms involved in central sympathetic overactivation and neurogenic hypertension development [29]. The results of our study suggest that the ISIAH rats are genetically predisposed to develop the oxidative stress in the brain stem. As neuronal chronic oxidative stress is known to play a key role in neurohumoral activation in hypertension, obesity and heart failure [30], we may suggest that the oxidative stress in the ISIAH brain stem may be one of the key factors contributing to stress-sensitive hypertension development in these rats.

The ISIAH rats were earlier characterized as a hypertensive strain with enhanced responsiveness to stressful stimulation [12]. It is worth to mention here three DEGs (*Gabra6*, *Gabrd*, and *Grm2*) encoding the proteins associated with neurotransmission (gamma-aminobutyric acid (GABA) A receptors and glutamate receptor). The expression of *Gabra6* and *Gabrd* was enhanced and the expression of *Grm2* was reduced in ISIAH brain stem. GABA and glutamate, respectively, are two major inhibitory and excitatory neurotransmitters in the adult mammalian brain [31], which have a direct impact on the sympathetic activity regulation [32]. An earlier study of the metabolic profile in the prefrontal cortex and hypothalamus of 3-month-old ISIAH and WAG rats revealed interstrain differences in the ratio of excitatory and inhibitory brain metabolites, including GABA and glutamate [33]. Based on changes in the expression of the genes *Gabra6, Gabrd* and *Grm2*, in common with the metabolomic data, we can assume that there may be changes in the brain stem of ISIAH rats associated with the regulation of sympathetic activity.

To identify the DEGs associated with hypertension and making the most contribution to inter-strain differences, we employed the approach based on the PLS regression, which is considered as an appropriate method for biomarker selection in metabolomic [34] and gene expression [35] studies. Among the DEGs that make highest impact to the inter-strain variations we have emphasized genes that are known to be associated with hypertension. These DEGs (*F2r, Chi3l1, Ephx2*, and *Tlr3*) are also associated with CNS diseases (see Table 2) and, in our opinion, deserve priority in further research directed to elucidation of the molecular mechanisms underlying the stress-sensitive hypertension. Their possible role in the disease development in ISIAH rats is discussed below.

F2r is a coagulation factor II (thrombin) receptor (protease activated receptor-1, PAR1) involved in regulation of the thrombotic response. It plays a role in blood coagulation, inflammatory response, and cell proliferation [36, 37]. It is expressed in multiple cell types in the CNS, with the most prominent expression in glial cells [38]. Activation of PAR1 increases the intracellular Ca^2+^ concentration both in neurons and in non-neuronal cells. This may contribute to the regional tissue circulation of the brain by the contraction of smooth muscle cells of small cerebral arterioles [39]. PAR1 activation enhances excitatory synaptic transmission secondary to the release of glutamate from astrocytes following activation of astrocytically-expressed PAR1 [38]. So, the enhanced transcription of *F2r* may play a key role in blood pressure regulation in ISIAH rats.

The protein encoding by *Chi3l1* gene is an inflammatory marker associated with insulin resistance and having a role in endothelial dysfunction and atherosclerosis [40]. It contributes to stimulation of angiogenesis of endothelial progenitor cells [41]. The increased expression of this gene in the brain stem (in nucleus tractus solitarii) of spontaneously hypertensive rats (SHR) was considered as a sign of NTS inflammatory state in SHR rats [42]. However, the expression of *Chi3l1* in ISIAH brain stem was found to be decreased as compared to control WAG rats. This discrepancy underlines the existence of the differences in molecular mechanisms of hypertension development in SHR and ISIAH rats.

The expression of *Ephx2* gene encoding soluble epoxide hydrolase (sEH) was strongly enhanced in the brain stem of ISIAH rats as compared to normotensive controls. These results are consistent with those reported for SHR rats [43]. The impact of the sEH overexpression to hypertension is well known. It was shown that sEH inhibitor 12-(3-adamantan-1-yl-ureido)-dodecanoic acid (AUDA) has renal and cardiovascular protective actions, lowers BP and prevents the development of salt-sensitive hypertension [44, 45]. In the brain stem of stroke-prone spontaneously hypertensive rats the epoxide hydrolase inhibitor reduced ischemic cerebral infarct size [46]. However, sEH inhibition by intracerebroventricular delivery of AUDA caused an increase of BP and heart rate in SHR rats [43]. So, the enhanced expression of epoxide hydrolase seems to play important role in the functioning of brain stem in hypertensive rats, however this role is not fully understood.

In our study *Ephx2* gene was one of 5 DEGs associated with arachidonic acid metabolism. Arachidonic acid (AA) is the predominant precursor for a family of lipid mediators [47]. In the brain stem of ISIAH rats, we have found the decreased expression of *Pla2g3* gene involved in release of AA and the downregulation of several genes involved in AA oxidative metabolism. Particularly, the decreased expression of *Ptgds* may lead to reduced amount of prostaglandin D2 (PGD2), which functions as a neuromodulator and as a trophic factor in the CNS. Besides, it is involved in smooth muscle contraction/relaxation processes, and is considered as a potent inhibitor of platelet aggregation. Another AA derivatives, epoxyeicosatrienoic acids (EETs), are produced by the action of cytochrome p450s. The downregulation of *Cyp2j10* may cause the reduced amount of EETs, which contribute to modulation of ion transport and gene expression, have an impact to vasorelaxation, and may exert an anti-inflammatory and pro-fibrinolytic effects [48]. The overexpression of *Ephx2* also may reduce the amount of EETs as they are hydrolyzed by sEH [47]. So, the changes in the arachidonic acid metabolic pathways may have significant impact to ISIAH brain stem function, and the *Ephx2* gene may be considered as a key one as its strong contribution to the inter-strain differences was also shown in the studies of hypothalamus, adrenal gland and kidney of the ISIAH and WAG rats [49–52].

The protein encoded by *Tlr3* gene is a member of the toll-like receptor family. The current study demonstrated that *Tlr3* is upregulated in brain stem of ISIAH rats. Earlier it was shown that TLR3 expression may be stimulated to modulate cell-cell and cell-matrix contacts of perivascular cells playing a role in angiogenesis and blood vessels permeability [53].

The work showed that altered expression of genes may have a role both in disease development and in compensatory processes. We may suggest that the genetic selection for many generations could result in gathering of the specific alleles of many genes contributing to the stress-sensitive hypertension in ISIAH rats. Our previously published results of the comparative study of single nucleotide polymorphisms (SNPs) in ISIAH and other hypertensive rat strains demonstrated the presence of multiple SNPs specific for ISIAH transcriptome and probably contributing to stress-sensitive hypertension development [54]. Among the genes that were identified in this work as candidates for future validation studies, several SNPs in mRNA of *F2r,* and *Tlr3* genes were found (Additional file 7: Table S7). As can be seen from the Additional file 7: Table S7, one of the non-synonymous mutations (c.2650A > G, p.Lys884Glu) in the *Tlr3* gene according to the annotation in the SIFT program (Sorting Intolerant From Tolerant, http://sift.bii.a-star.edu.sg/) may affect the function of the protein. This SNP was also found in the genomes of the ACI/N and ACI/EurMcwi rats, which are models of chronic kidney disease, as well as in the BBDP/Wor rats that are prone to developing diabetes (RGD, http://rgd.mcw.edu/) [55].

In several studies analyzing human populations, it was shown that polymorphisms in or near the *EPHX2* gene may contribute to the risk of ischemic stroke [56–58]. Polymorphisms in the coding and regulatory regions of the *Ephx2* gene that can affect the expression level and activity of sEH have also been described in SHR rats with spontaneous hypertension [59, 60].

An increased level of *Ephx2* transcription was found in several tissues/organs (brain stem, hypothalamus, adrenal gland and kidney) of ISIAH rats compared to WAG rats [49–52]. However, SNPs were not identified in the *Ephx2* mRNA sequence in ISIAH rats. This allows us to assume that the sEH protein function in ISIAH rats is not changed, and the highly reliable increase in the transcription level of the *Ephx2* gene in all analyzed tissues/organs of ISIAH rats may be related to the sequence features of the promoter region of this gene, or to the features of the signal regulation mechanisms of its expression. The second assumption can be based on the data we have on the localization of the *Ephx2* gene in the genetic locus (QTL) on chromosome 15, which we previously associated with plasma corticosterone concentrations under the influence of emotional stress [61], as well as on data that the level of transcription of the *Ephx2* gene significantly increases under the influence of emotional stress, which was shown when studying the level of its transcription in the renal medulla of ISIAH rats [62].

Many researchers are inclined to believe that sEH encoded by the *Ephx2* gene can be considered a promising target for pharmaceutical effects aimed at treating hypertension [63, 64], cardiovascular diseases [65], cerebral ischemia [66], as well as cerebral stroke [67].

Taking our results into account, we would like to conclude that the study revealed multiple DEGs in brain stem of hypertensive ISIAH and normotensive WAG rats and underlined the most important biological processes and pathways related to stress-sensitive hypertension. These results confirmed the complex nature of the pathogenesis of hypertension in the ISIAH rats, which was earlier demonstrated in the comparative studies of transcriptional profiling of other target tissues/organs (hypothalamus, adrenal gland and kidney) from ISIAH and WAG rats [49–52]. Gene *Ephx2* may be considered as a major candidate gene, as it was defined as one, making the most contribution to the inter-strain differences not only in the brain stem of ISIAH rats but also in all other analyzed tissues/organs (hypothalamus, adrenal gland and kidney) known as targets in hypertension development [49–52].

Over the past decade, the development of high-throughput sequencing of genomes and transcriptomes has contributed significantly to understanding the molecular basis of polygenic diseases. RNA-Seq is one of the effective methods for comparative analysis of transcriptomes [68]. Due to the fact that the results of RNA-Seq analysis show high levels of reproducibility for both technical and biological replicates [69, 70], a small number of repetitions that are commonly used in the analysis, seems to be not a critical limitation of the method. In our work, we found a high correlation (r = 0.98) between the qPCR and RNA-Seq data, which is similar to the results obtained by other researchers [69]. Anyway, we would like to discuss an important point. When performing RNA-Seq, the list of differentially expressed genes is compiled in accordance with some generally accepted criteria for processing statistical data. The use of statistically significant confidence levels is common practice when performing various experimental works. However, we would like to point out the fact that genes that just slightly fall short of the accepted threshold of statistically significant q value < 5% are out of the researchers’ field of vision. Given that not many samples of animals were used in our work, we assume that some of the functionally important genes for random reasons could not be included in the list of differentially expressed genes. Therefore, in Additional file 8: Table S8 we provide a list of genes, the interstrain differences in the expression level of which were characterized by *P* values < 0.001, but which did not fall into the main list of differentially expressed genes.

The data obtained in the current study are useful for better understanding of the genetic mechanisms underlying the complexity of the brain stem processes in stress-sensitive form of hypertensive disease and for identifying the brain molecular determinants of arterial hypertension, which have a potential to be used as therapeutic targets for pharmacological intervention.

## Methods

### Animals

The male rats of hypertensive ISIAH/Icgn (Inherited Stress Induced Arterial Hypertension) and normotensive WAG/GSto-Icgn (Wistar Albino Glaxo) strains were used in the study. Both rat strains were derived from an outbred stock of Wistar rats independently of each other. However, WAG rats are normotensive and are used in the present experiment as a control strain. All rats were bred in the Center for Genetic Resources of Laboratory Animals at the Institute of Cytology and Genetics, Siberian Branch of the Russian Academy of Sciences, (Novosibirsk, RF). Rats were housed under standard conditions with free access to food and water. The 3-month old rats were decapitated and their brain stems (medulla oblongata and pons) were isolated and stored in RNA Later (Qiagen, Chatsworth, CA) at -70 °C until use. Animal experiments were conducted according to the protocols approved by the Institutional Animal Care and Use Committee (approval documentation number 41).

### RNA-Seq analysis

The frozen brain stem samples from ISIAH (*n* = 3) and WAG (n = 3) male rats were sent to JSC Genoanalytica (Moscow, Russia) company specializing in conducting RNA-Seq analysis, where RNA-Seq based transcriptome profiling was performed. Dynabeads mRNA Purification Kit (Ambion, USA) was used to extract the mRNA. cDNA libraries were constructed using NEBNext mRNA Library Prep Reagent Set for Illumina (NEB, USA). All experimental procedures were performed according to the manufacturer’s protocols. cDNA libraries were single-end sequenced on an Illumina Hiseq 1500 platform (Illumina Sequencing, San Diego, USA) with read length of 50 bases. All samples were analyzed as biological replicates. The adapter trimming and low-quality sequence removal were done before the mapping of the sequencing data to the Rat Genome Sequencing Consortium (RGSC) Rnor_5.0\rn5 reference genome with the use of Tophat2 (v2.0.13) aligner [71]. The quality metrics of the mapped data were collected (Additional file 9: Table S9) using CollectRnaSeqMetrics from the Picard tools suite (v2.4.1) (http://broadinstitute.github.io/picard/).

Cufflinks/Cuffdiff (v2.1.1) programs were run to evaluate gene expression levels in FPKM (fragments per kilobase of transcript per million mapped reads) and to identify the DEGs. Annotation of genes was based on NCBI Gene/RefSeq database. A gene was considered to be expressed (transcribed) if it successfully passed statistical testing in the Cufflinks program and was given ‘OK’ status. Genes were considered as differentially expressed at a false discovery rate (FDR) < 5% [72]. Heatmap and cluster analysis were performed in R environment using hierarchical complete-linkage method and Euclidean distance metric. The RNA-Seq data were deposited in NCBI Sequence Read Archive (SRA) under the accession number PRJNA299102 (https://www.ncbi.nlm.nih.gov/bioproject/?term=PRJNA299102). These data were used as a part of the previously published study directed to the identification of the ISIAH strain specific SNPs [54]. The SNP analysis described 6 biological replicates for brain stems for each (ISIAH and WAG) rat strain. 3 of these were those described in the current manuscript and another 3 biological replicates were the results of the RNA-Seq analysis from brain stems of 1 month old prehypertensive ISIAH and control WAG rats, which can’t be combined with the data reported in here because of the age related effects on transcriptomes.

Functional annotation

The functional annotation of DEGs was performed by DAVID (The Database for Annotation, Visualization and Integrated Discovery) gene annotation tool (http://david.abcc.ncifcrf.gov/) [73, 74]. The *Rattus norvegicus* genome was utilized as the background list for the over-representation analysis. The Gene Ontology (GO) option in DAVID and Kyoto Encyclopedia of Genes and Genomes (KEGG, http://www.genome.jp/kegg/) Pathway Database were used for the identification of the significantly (*p* < 0.05) enriched biological processes and metabolic pathways. Rat Genome Database (RGD, http://rgd.mcw.edu/) was employed to define the DEGs associated with hypertension and central nervous system (CNS) diseases. Atlas of combinatorial transcriptional regulation in mouse and man [75], and Panther classification system (http://www.pantherdb.org/) [76] were used to reveal the DEGs encoding the transcription factor genes.

### Quantitative real time PCR (qPCR)

Validation of differential expression of several selected genes was performed by qPCR. The details of this procedure have been described earlier [49]. The TRI reagent (Molecular research center, USA) was used to isolate the total RNA, which then was treated with DNase I (Promega, USA) for removing the residual genomic DNA. Reverse transcription was performed using the reverse transcription buffer (Vektor-Best, RF), dNTPs, random nonanucleotide primers (Biosan, RF), MoMLV (Vektor-Best, RF), and 3 μg of total RNA. cDNA synthesis was performed using the following protocol: 1 h at 37 °C, 30 min at 42 °C, 10 min at 50 °C, and 5 min at 75 °C to inactivate the enzyme.

qPCR was carried out in a final volume of 20 μl. The reaction mixture contained a master mix with SYBR Green (Vektor-Best, RF), 0.15 mM of each forward and reverse primers, the cDNA template, and 1 unit of HotStart Taq polymerase (Vektor-Best, RF). The following target genes were used chitinase 3-like 1 (*Chi3l1*)*,* cytochrome P450, family 11, subfamily b, polypeptides 1 & 2 (*Cyp11b1* and *Cyp11b2),* epoxide hydrolase 2, cytoplasmic (*Ephx2*)*,* coagulation factor II (thrombin) receptor (*F2r*)*,* and toll-like receptor 3 (*Tlr3*)*.* The ribosomal protein L30 (*Rpl30*) was used as endogenous control for *Cyp11b1, Cyp11b2 and Ephx2* genes and peptidylprolyl isomerase A (*Ppia*) was used as endogenous control for *Chi3l1, F2r* and *Tlr3* genes. Primer sequences and their characteristics are shown in Additional file 10: Table S10.

qPCR was run in a iCycler iQ4 Real-Time PCR Detection System (Bio-Rad Laboratories, USA). The reaction started at 94 °C for 1 min was followed by 40 cycles of 15 s at 94 °C, 20 s at primers annealing temperature (see Additional file 10: Table S10), 20 s at 72 °C. The melting curve was generated in the range of 65 °C to 94 °C. Standard-curve quantitation method was employed to determine the relative levels of transcripts [77]. To get the standard cDNA, the aliquots from each of the synthesized cDNA samples were pooled. In each experiment, cDNA samples with primers for the target gene (four repeats per cDNA sample), the same samples with primers for the reference gene (four repeats), and standard dilutions of cDNA (1: 1, 1: 4, 1: 16, 1: 64) with primers for the target gene (two repeats) and with primers for the reference gene (two repeats) were placed on the same plate. The relative amount of the tested cDNA was determined using calibration curves built by the iCycler iQ4 Real-Time PCR Detection System software. The value obtained for the target gene was then normalized to the value detected for the reference gene.

The significance of differences between the means of control and experimental samples was estimated by Student’s t-test. Data were expressed as mean and standard error of mean (M ± SEM). A *P*-value of < 0.05 was chosen as the criterion for statistical significance.

#### Determining genes driving inter-strain differences

The acquired RNA-Seq data sets (FPKM values) were log transformed, standardized (by subtracting the mean and dividing by the standard deviation), and scaled using the principal coordinates (PCO) method based on Euclidean metric distances. Then the partial-least squares discriminant analysis (PLS-DA) was employed using the pattern of co-variation for linear combinations between two blocks of variables [78]. These procedures resulted in the construction of the PLS-DA Axes maximizing the distances between hypertensive and normotensive rats. The following calculation of the Pearson correlation coefficients helped to detect a set of variables (expressed genes) that are expected to maximize the covariance between fixed dummy matrix representing group membership for ISIAH and WAG rats and gene expression in these rats. The calculated correlation revealed the genes characterized by the most deviation along the first functionally meaningful synthetic axis (PLS-DA Axis 1). These genes may be considered as candidates making the most contribution to inter-strain differences.

## Additional files

Additional file 1: **Table S1.** Genes differentially expressed in brain stems of 3-month old hypertensive ISIAH and normotensive control WAG rats. (XLSX 30 kb)

Additional file 2: **Figure S2.** Heatmap of the DEGs (JPG 4887 kb)

Additional file 3: **Table S3.** The genes with the detected expression in brain stem of only one rat strain (XLSX 9 kb)

Additional file 4: **Figure S4.** GO terms for biological processes (JPG 2192 kb)

Additional file 5: **Table S5.** Differentially expressed genes in GO term groups (XLSX 73 kb)

Additional file 6: **Table S6.** KEGG metabolic pathways (XLSX 13 kb)

Additional file 7: **Table S7.** SNPs in candidate genes (XLSX 459 kb)

Additional file 8: **Table S8.** Additional list of genes which are differentially expressed at *P* value< 0.001 (XLSX 37 kb)

Additional file 9: **Table S9.** The summary statistics for the sequenced libraries (XLSX 15 kb)

Additional file 10: **Table S10.** Primers used in qPCR (DOCX 12 kb)

## Abbreviations

AA: arachidonic acid
AUDA: 12-(3-adamantan-1-yl-ureido)-dodecanoic acid
BP: blood pressure
CNS: central nervous system
DAVID: Database for Annotation, Visualization and Integrated Discovery
DEG: differentially expressed genes
EETs: epoxyeicosatrienoic acids
FPKM: fragments per kilobase of transcript per million mapped reads
GABA: gamma-aminobutyric acid
GO: Gene Ontology
ISIAH: Inherited Stress Induced Arterial Hypertension
KEGG: Kyoto Encyclopedia of Genes and Genomes
NO: nitric oxide
NTS: nucleus tractus solitarii
PCO: principal coordinates method
PLS-DA: partial-least squares discriminant analysis
qPCR: Quantitative real time PCR
QTL: quantitative trait loci
RGD: Rat Genome Database
RGSC: Rat Genome Sequencing Consortium
RNA-Seq: RNA sequencing
ROS: reactive oxygen species
RVLM: rostral ventrolateral medulla
sEH: soluble epoxide hydrolase
SHR: spontaneously hypertensive rats
SNPs: single nucleotide polymorphisms
VSM: vascular smooth muscle
WAG: Wistar Albino Glaxo
