# Supplementary figures and images for: The differences in brain stem transcriptional profiling in hypertensive ISIAH and normotensive WAG rats
Source: BMC Genomics. 2019 May 8;20(Suppl 3):297. doi: 10.1186/s12864-019-5540-5 (PMC7226933; doi:10.1186/s12864-019-5540-5)

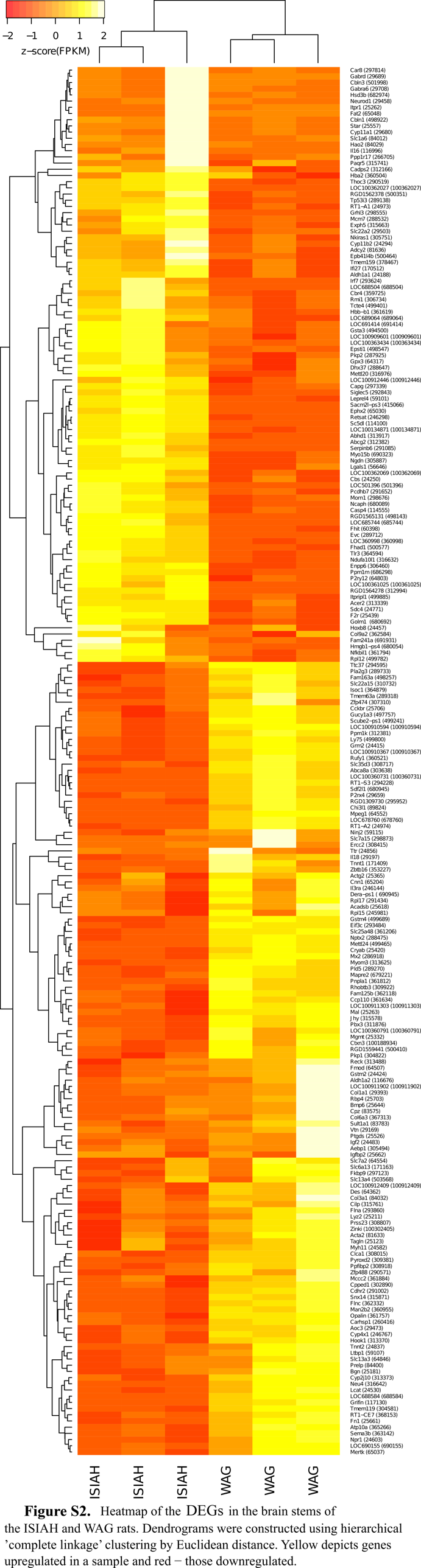

Supplement: Supplementary file 2 — Figure S2. Heatmap of the DEGs (JPG 4887 kb) [file 12864_2019_5540_MOESM2_ESM.jpg]

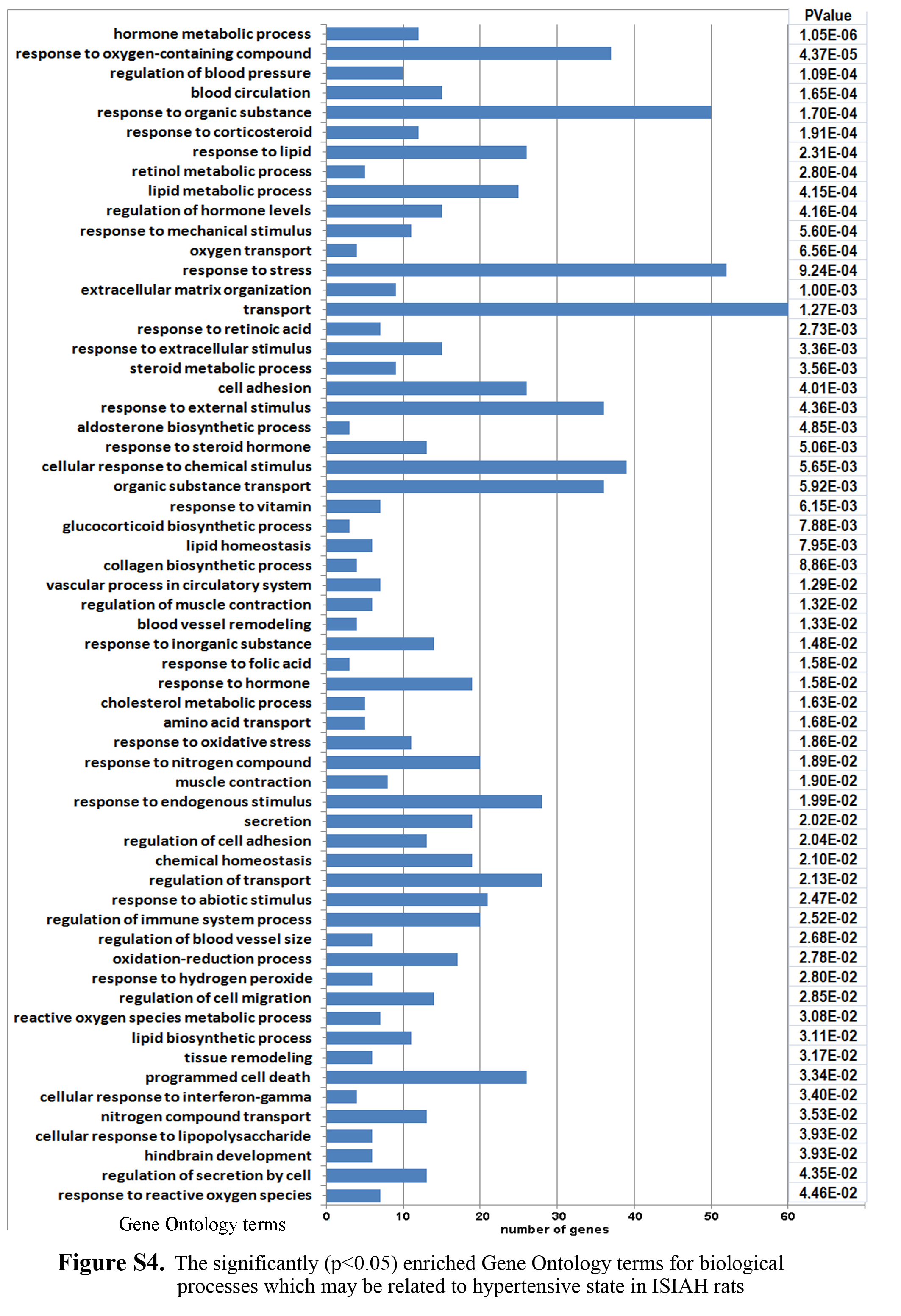

Supplement: Supplementary file 4 — Figure S4. GO terms for biological processes (JPG 2192 kb) [file 12864_2019_5540_MOESM4_ESM.jpg]
