# Supplementary material for: The differences in brain stem transcriptional profiling in hypertensive ISIAH and normotensive WAG rats
Source: BMC Genomics. 2019 May 8;20(Suppl 3):297. doi: 10.1186/s12864-019-5540-5 (PMC7226933; doi:10.1186/s12864-019-5540-5)
Supplement: Supplementary file 10 — Table S10. Primers used in qPCR (DOCX 12 kb) [file 12864_2019_5540_MOESM10_ESM.docx]

| Gene symbol | primers, 5’--->3’ | | Length of PCR fragment,  bp | Annealing temperature,  ^o^C |
| --- | --- | --- | --- | --- |
|  | forward | reverse |  |  |
| ***Chi3l1*** | CTCTCAAGACCAGAAACCCC | GATCCAGTCCATCAAAGCCA | 165 | 62 |
| ***Cyp11b1*** | CATGGAAGCCAGCCATTTTGT | GTGAATGTCACGCTCTCAGGT | 84 | 62 |
| ***Cyp11b2*** | TGTGGCAGCACTAATAACTCA | GCCAGCTCAAAAAGGGTCA | 126 | 62 |
| ***Ephx2*** | TTTCTTGGAGGTACCAGATCC | CAGTCATGGCCAATGAACAC | 193 | 62 |
| ***F2r*** | CTTCCCGCGTTCCTATGAGA | TTAAGTAGACTGCCCTGCCC | 183 | 62 |
| ***Ppia*** | TTCCAGGATTCATGTGCCAG | CTTGCCATCCAGCCACTC | 206 | 62-64 |
| ***Rpl30*** | CATCTTGGCGTCTGATCTTG | TCAGAGTCTGTTTGTACCCC | 143 | 61-64 |
| ***Tlr3*** | AGAAGACGCTACAGCTTTCC | TGCAGTCAGCTACGTTGTAT | 207 | 63 |

Table S10. Primers used in qPCR
